# Three-dimensional printing-guided percutaneous transcatheter replacement in coarctation of the aorta: a retrospective study

**DOI:** 10.3389/fcvm.2024.1429470

**Published:** 2024-12-13

**Authors:** Qing He, Chennian Xu, Yang Liu, Ping Jin, Mengen Zhai, Rui Qiao, Zhiyuan Tian, Bin Cui, Jian Yang

**Affiliations:** ^1^ Department of Cardiovascular Surgery, Xijing Hospital, Air Force Medical University, Xi’an, Shaanxi, China; ^2^ Key Laboratory of Gastrointestinal Pharmacology of Chinese Materia Medica of the State Administration of Traditional Chinese Medicine, Department of Pharmacology, School of Pharmacy, Air Force Medical University, Xi’an, Shaanxi, China; ^3^ Department of Cardiovascular Surgery, General Hospital of Northern Theater Command, Shenyang, Liaoning, China; ^4^ Department of 1st Cadre Ward, General Hospital of the Northern Theater Command, Shenyang, Liaoning, China; ^5^ The 79th Group Military Hospital of the Chinese People’s Liberation Army, Liaoyang, Liaoning, China

**Keywords:** 3D printing, coarctation of aorta, covered stent, interventional therapy, transcatheter replacement

## Abstract

**Background:**

To evaluate the feasibility, effectiveness and assistant effect of 3D printed aortic model in the treatment on congenital coarctation of the aorta (CoA) in adolescents and adults.

**Methods:**

From December 2018 to December 2023, a total of 10 patients with congenital coarctation of aorta underwent percutaneous balloon dilatation covered stent implantation in the department of cardiovascular surgery, Xijing Hospital. There were 6 males and 4 females whose average age was (27.68 ± 13.45) years. One case complicated with ventricular septal defect (VSD). The CT data of aorta were collected before operation, and the aorta was reconstructed by Mimics software and printed with 3D printing technology. The operation simulation was performed before operation to determine the best operation plan. The clinical data during hospitalization and follow-up were analyzed.

**Results:**

All the patients of CoA were successfully treated with percutaneous balloon dilatation covered stent implantation. The narrowest average diameter of CoA increased from (4.35 ± 2.61) mm before operation to (16.84 ± 1.99) mm immediately after operation (*P* < 0.05). The mean transconstrictive systolic pressure difference decreased from (81.29 ± 18.72) mmHg before operation to (15.52 ± 7.47) mmHg after operation (*P* < 0.05). The mean systolic blood pressure of the right upper limb decreased from (182.05 ± 38.99) mmHg preoperatively to (141.95 ± 32.11) mmHg postoperatively (*P* < 0.05). The mean systolic blood pressure of the lower limb increased from (121.52 ± 27.84) mmHg preoperatively to (131.81 ± 32.39) mmHg postoperatively (*P* < 0.05). Two patients with PDA and VSD underwent interventional occlusion at the same time without shunt. During the period of hospitalization and follow-up, there were significant cardiovascular complications.

**Conclusions:**

Percutaneous balloon dilatation covered stent implantation is effective in the treatment of adolescents and adults with CoA in the short and medium term, with fewer complications, and the long-term effect needs furthshdie1er study.

## Introduction

Coarctation of the aortic arch (CoA) usually occurs at the descending part of the aortic arch, the distal end of the left subclavian artery, and the junction of the ductus arteriosus. It has become a common congenital heart anomaly in clinical practice, accounting for 5%–8% of congenital heart disease (CHD) (1, 2). Commonly seen in males, with a male to female ratio of 3.5:1, it can exist alone, and cardiac surgery can also cause aortic constriction. More than 50% of patients often have other congenital cardiovascular malformations, mainly including patent ductus arteriosus, ventricular septal defect, and bivalve aortic valve. In clinical practice, for systolic arterial pressure gradient greater than 20 mmHg (1 mmHg = 0.133 kPa). Congenital coarctation of the aorta can be diagnosed by combining with corresponding imaging changes (1, 2). However, the surgery for CoA was complicated and the mortality and complication rate were high. In recent years, percutaneous balloon dilatation covered stent implantation is a new technology for the treatment of CoA (3, 4). With the continuous improvement of surgical techniques, the mortality rate has decreased (5). In recent years, with the continuous development of intervention technology, intervention therapy has made rapid progress in the treatment of congenital heart disease. Especially in the past decade, NuMED stents and balloon in balloon (BIB, NuMed Corporation, USA) catheters specifically designed for the treatment of vascular obstructive diseases related to congenital heart disease have increasingly attracted the attention of clinical doctors (6, 7).

Structural heart disease (SHD) mediates Treatment requires a deep understanding of perioperative cardiac pathophysiology and interventional instruments, and traditional imaging examinations are no longer sufficient to meet these requirements. There are still shortcomings in the development of specialized instruments, medical education, medical imaging, and nursing cooperation in transcatheter intervention surgery, and there are also differences in understanding the 3D, 4D and artificial intelligence (8). Futhermore, studys have indicated that the application of CFD simulations may be useful for blood flow reconstruction for clinical conditions in circulatory system (9, 10). In order to bridge the gap between virtual reality and integrate the advantages of computer science and biomedical engineering, 3D printing, computer modeling, and artificial intelligence (AI) may play their advantageous roles. Our research team has applied 3D printing technology for preoperative multimodal imaging evaluation and *in vitro* simulation in cardiovascular intervention surgeries such as TAVR, TMVR, PVL, etc., and has achieved good results in assisting in the formulation of intervention surgical plans and physician training. Similarly, we would like to conduct an application study using 3D printing technology for preoperative evaluation and simulation of aortic arch constriction covered stent implantation, in order to further improve the role of 3D printing technology in preoperative imaging evaluation of structural heart disease.

## Materials and methods

### General information of patients

**Figure 1:**
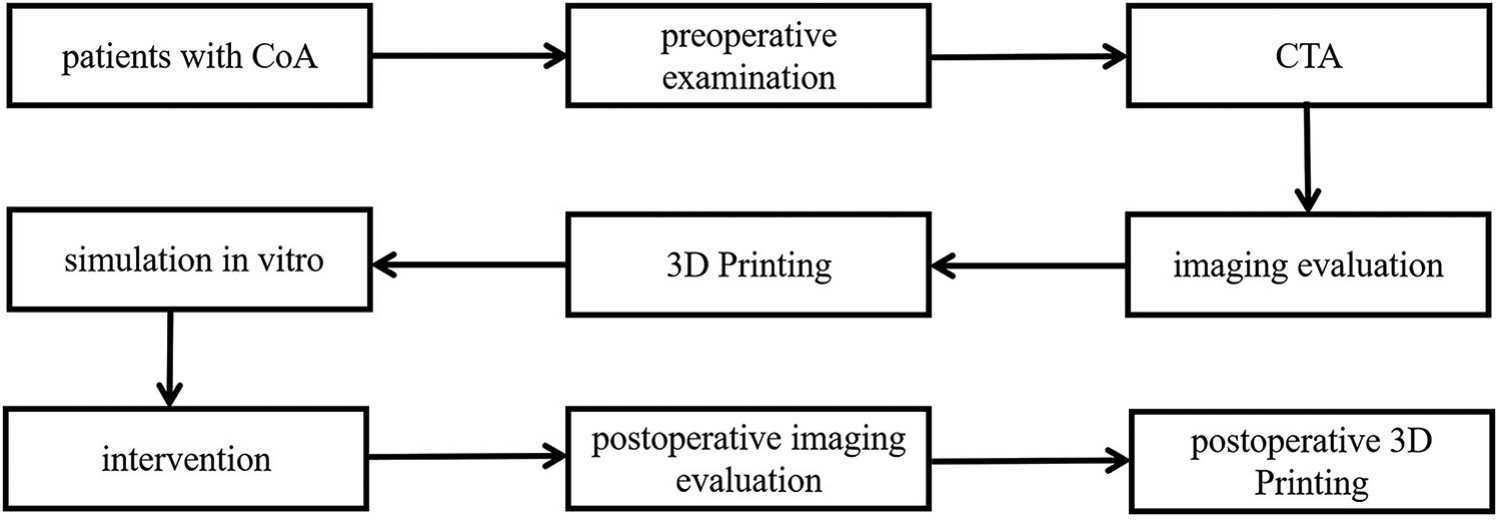
Protocal of the experiment.

A total of 10 patients with CoA underwent percutaneous balloon expandable covered stent implantation in Cardiovascular Surgery, Xijing Hospital from December 2018 to December 2023 were enrolled. Among them, there were 6 males and 4 females whose average age was (27.68 ± 13.45) years. Routine arterial blood pressure measurements showed that patients had different degrees of hypertension (right upper limb), and lower limb blood pressure was generally lower than upper limb blood pressure by 20 mmHg or more. There were 2 cases with ventricular septal defect and 2 cases with patent ductus arteriosus. All patients underwent percutaneous human-to-human occlusion at the same time. One case had mild aortic insufficiency. Preoperative ECG, echocardiography and aortic CTA were performed to confirm the clinical diagnosis. Routine preoperative biochemical examinations were performed to assess the patient's condition. All patients were diagnosed with CoA for the first time and none undergo plain angioplasty before stent placement. All selected patients have obtained the informed consent of their patients and their families, and signed the informed consent form for surgery (Figure 1).

### 3D Printing and preoperative simulation *in vitro*

**Figure 2:**
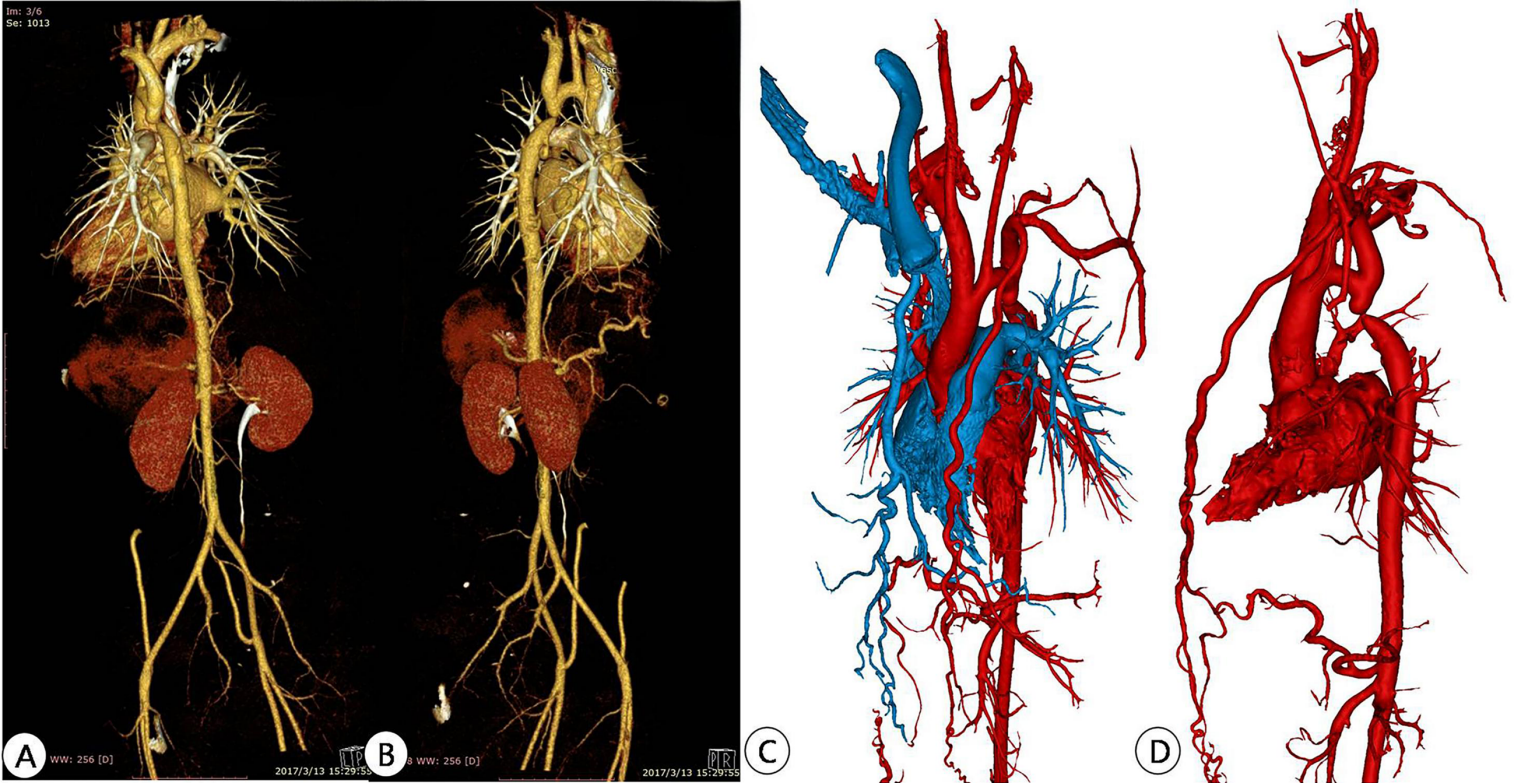
Preoperative CTA reconstruction image of the patient. **(A,B)** CTA reconstruction imaging shows the location of aortic arch constriction; **(C,D)** Using computer reconstruction technology, segment the anatomical structure around aortic constriction and highlight the location of aortic constriction.

**Figure 3:**
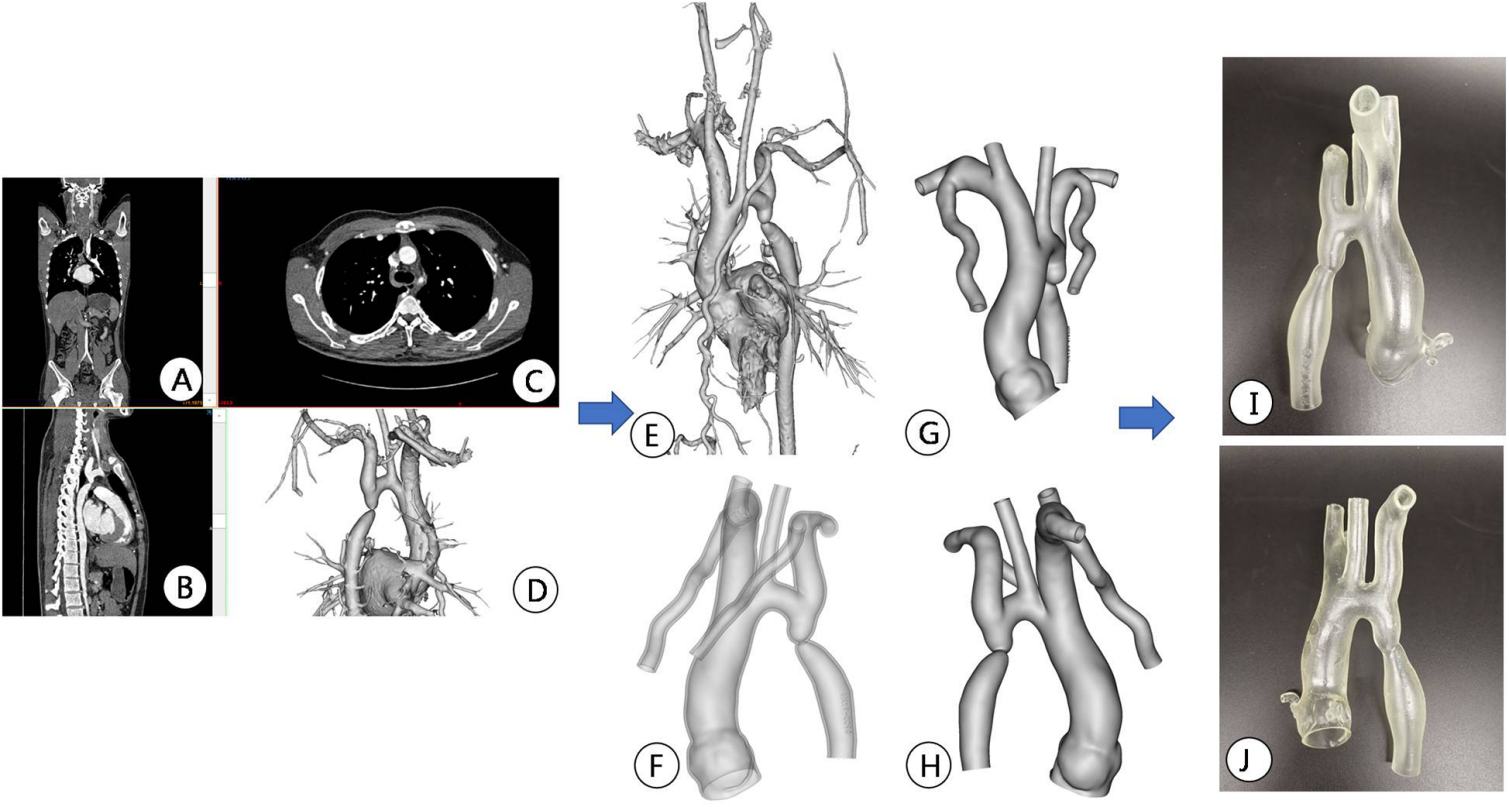
Preoperative CT image data reconstruction and 3D printing model of patients. **(A–C)** Display CTA images of the aorta in different sections; **(D,E)** Mimics software displays the location of active artery constriction at different angles after reconstruction; **(F–H)** segmentation, decoration, and reconstruction of images for subsequent 3D printing; **(I,J)** 3D printed aortic constriction model displayed on different sides.

**Figure 4:**
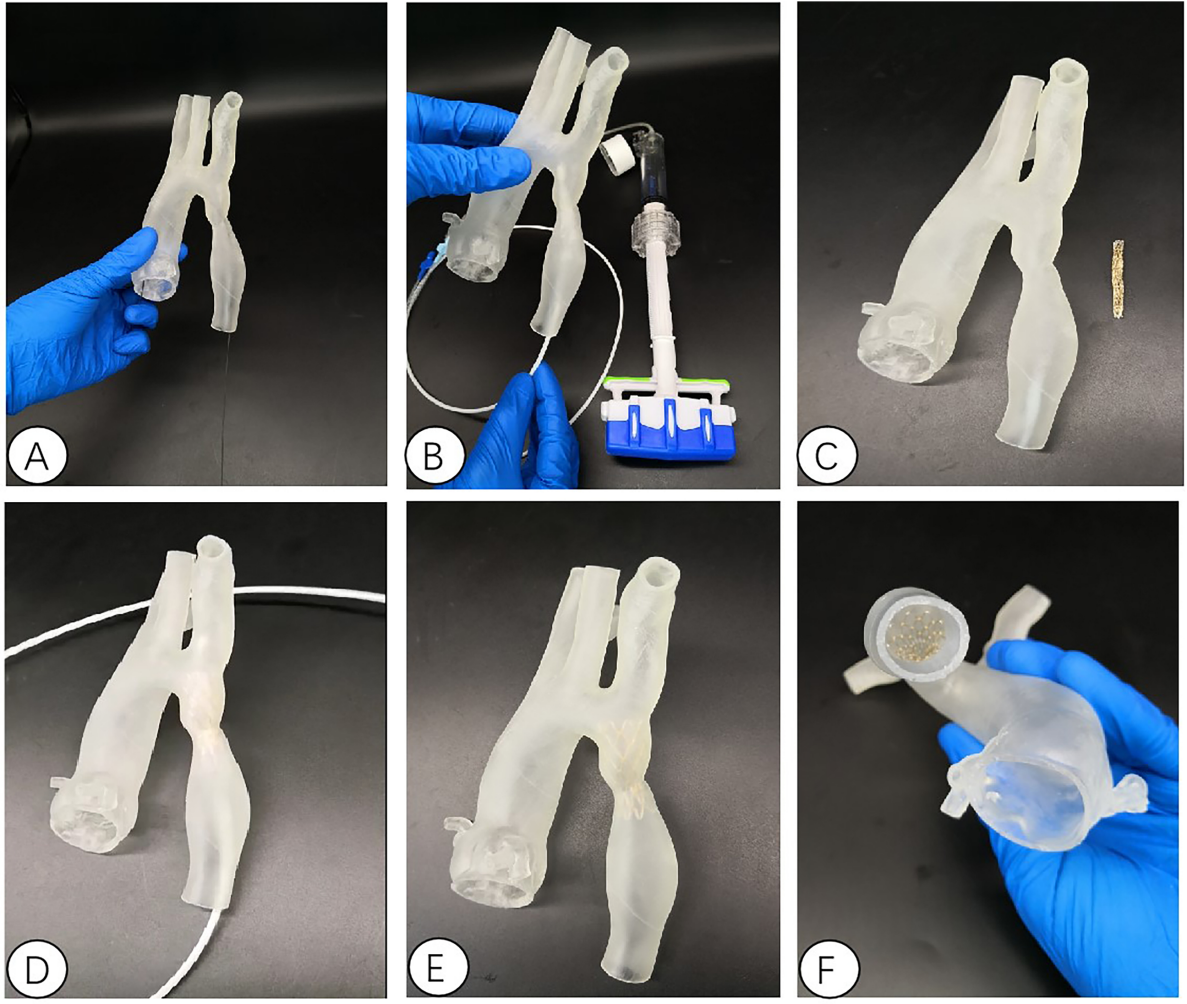
Interventional simulation using 3D printed models. **(A)** 3D printed aortic constriction model; **(B)** Using a 3D printing model of aortic constriction to simulate balloon dilation *in vitro*; **(C)** 3D printed aortic constriction model and stent with covered membrane; **(D)** *in vitro* simulated stent release at aortic constriction; The position relationship and fit degree between the **(E,F)** bracket and the 3D printed model after its release.

All patients underwent chest area scanning using Siemens Flash dual source CT machine (Siemens AG, Germany), with a scanning range from the aortic arch to the pubic symphysis, with a scanning layer thickness of 7.0 mm and a reconstruction layer thickness of 0.75 mm. A total of 100 ml of iopromide (300 mgI/ml, Bayer Schering Pharma AG, Germany) was injected through the elbow vein at a rate of 3.0 ml/s. Import CT scan data in DICOM (digital Imaging and Communications in Medicine) format into Mimics 21.0 (Materialise, Leuven, Belgium) software for whole heart and aortic reconstruction, with a focus on reconstructing the site of aortic constriction and adjacent vascular structures. The extraction range of printed model image data in the reconstructed data includes the whole heart and aorta, which are segmented and surface modified with surrounding tissues to form an STL (STereoLithography) format file and imported into Magic software for model surface modification. After that, the data is imported into a Form2 printer (Stratasys Corporation, USA), and the printing materials are mainly silicone and relatively hard transparent resin materials (Clear V4, Formlabs Corporation, USA), in order to fully simulate the structure of the patient's aorta and create a model of the patient's heart and aorta. Before intervention, a 3D printed model and a covered stent delivery system were used *in vitro* to conduct personalized analysis of the anatomical structure of the patient's heart and aortic constriction site. The procedure of the guide wire, catheter, and delivery system through the aortic constriction site was simulated, and the surgical process was simulated *in vitro*. The optimal position and angle for releasing the covered stent were determined, and a personalized surgical plan was developed (Figures 2–4).

### Procedure of intervention

**Figure 5:**
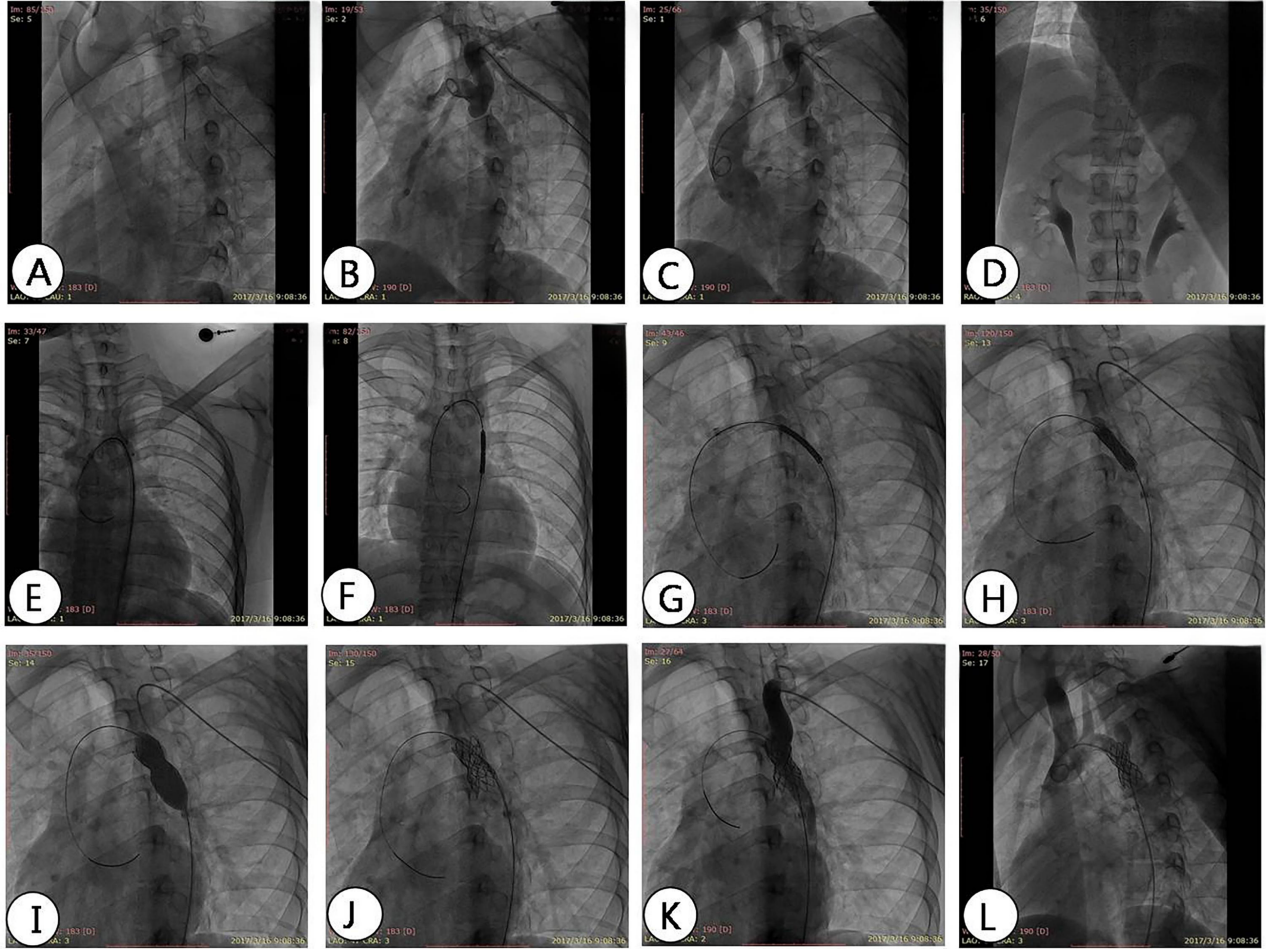
DSA angiography shows the process of stent implantation. **(A)** The contrast catheter passes through the constriction of the aorta; **(B)** angiography shows the location and degree of aortic arch constriction; **(C)** changing the angle of imaging to show narrowing of the aortic arch; **(D)** feeding into the conveying system from the femoral artery; **(E)** the conveying system; **(F–H)** transports the bracket to the predetermined position, narrows the plane, and performs imaging at different angles to determine the release position; **(I)** balloon dilation and release of stent; **(J)** evacuate the conveying system; **(K,L)** angiography shows the position of the covered stent and evaluates the pressure difference after expansion.

In the hybridization room, all patients were in supine position and operated under local anesthesia. Seldinger technique was used to puncture the left radial artery and the right femoral artery. The sheaths of 5F and 6F arteries were placed respectively. After intravenous administration of heparin (1 mg/kg), the pigtail catheter was placed into the ascending aorta via the left radial artery. The narrowing position, length, degree, proximal and distal aorta diameter, the relationship with the surrounding vessels and the presence of other vascular lesions were defined by multi-angle projection angiography. The right coronary artery catheter and the super slippery loach guide wire were placed through the right femoral artery. The guide wire catheter passed through the narrowing lesion to the ascending aorta. The aorta was measured before and after stenosis and the Lunderquist guide wire (Cook Medical Trading Co., Ltd., China) was exchanged. Cheatham-Platinum covered stent (CP stent, Shenzhen Lifetech Scientific Medical Equipment Co., Ltd., China) and balloon in balloon (BIB, NuMed Corporation, USA) catheter of suitable length were selected. Balloon and covered stent were assembled *in vitro*. Steel protective inner core was built into balloon, and outer sheath tube protective stent was pre-placed on BIB balloon respectively. The 12F transporting sheath tube was inserted along the Lunderquist guide wire, the inner core was removed, the guide wire was retained, and the selected BIB balloon catheter and CP stent system for aortic coarctation were fed into the 12F transporting sheath tube. After satisfactory positioning, the stent was released, and the contrast agent was pre-dilated by injecting a high-pressure surgical syringe through a red interface. The contrast agent was injected by a high-pressure hand syringe through a blue interface rapidly and fully. Extracorporeal balloon dilatation showed lumbar sign of stenosis and rapid withdrawal of syringe. Angiography and manometry were performed again to observe the position and shape of stent, and aortic pressure was measured before and after stenosis. After satisfactory pressure difference, the catheter was withdrawn. The femoral artery puncture point was sutured with Perclose ProGlide vascular suture (Abbott Company, USA) and compressed and bandaged (Figure 5).

### Postoperative treatment and follow-up

At least 3 months after the operation, the patient persisted in taking dual antiplatelet aggregation drugs orally. After 3, 6, 12 months to the hospital for review, 1 year after the annual outpatient follow-up. Ask patients about the improvement of general symptoms, measure the blood pressure of limbs, and review the color echocardiography, aortic CTA and 3D printing if necessary.

### Statistical analysis

All demographic, valve-related procedural and outcome data, and clinical and anatomic data were obtained from a retrospective review of patient charts and procedural records. Statistical analyses were conducted using SPSS 22.0 software (IBM SPSS Statistics for Macintosh, Version 22.0, IBM Corp, Armonk, NY). Continuous variables are presented as means ± SD and categorical variables are expressed as percentages. Univariable comparisons were performed with the Student unpaired *t*-test for continuous normally distributed data and the chi-square test was used for categorical data. Values of *P* < 0.05 were considered statistically significant.

## Results

**Figure 6:**
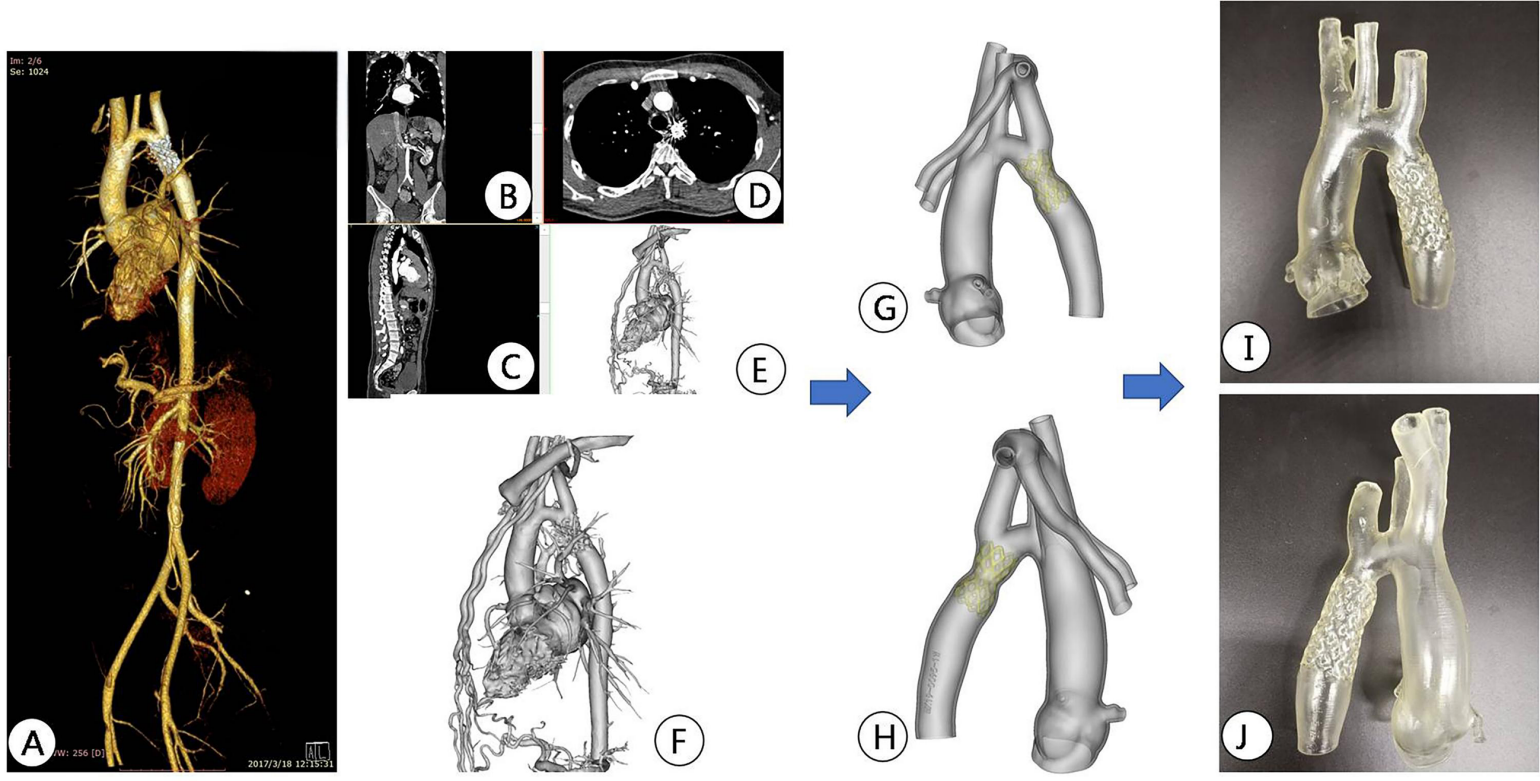
CT image reconstruction and 3D printing model after aortic coarctation surgery. **(A)** Postoperative aortic CTA reconstruction imaging showed stent position; **(B–D)** CTA images were imported into Mimics software and displayed different sections; **(E,F)** reconstruction images after stent implantation; **(G,H)** reconstructed images were segmented and modified for subsequent 3D printing; **(I,J)** 3D printed models after stent implantation.

**Figure 7:**
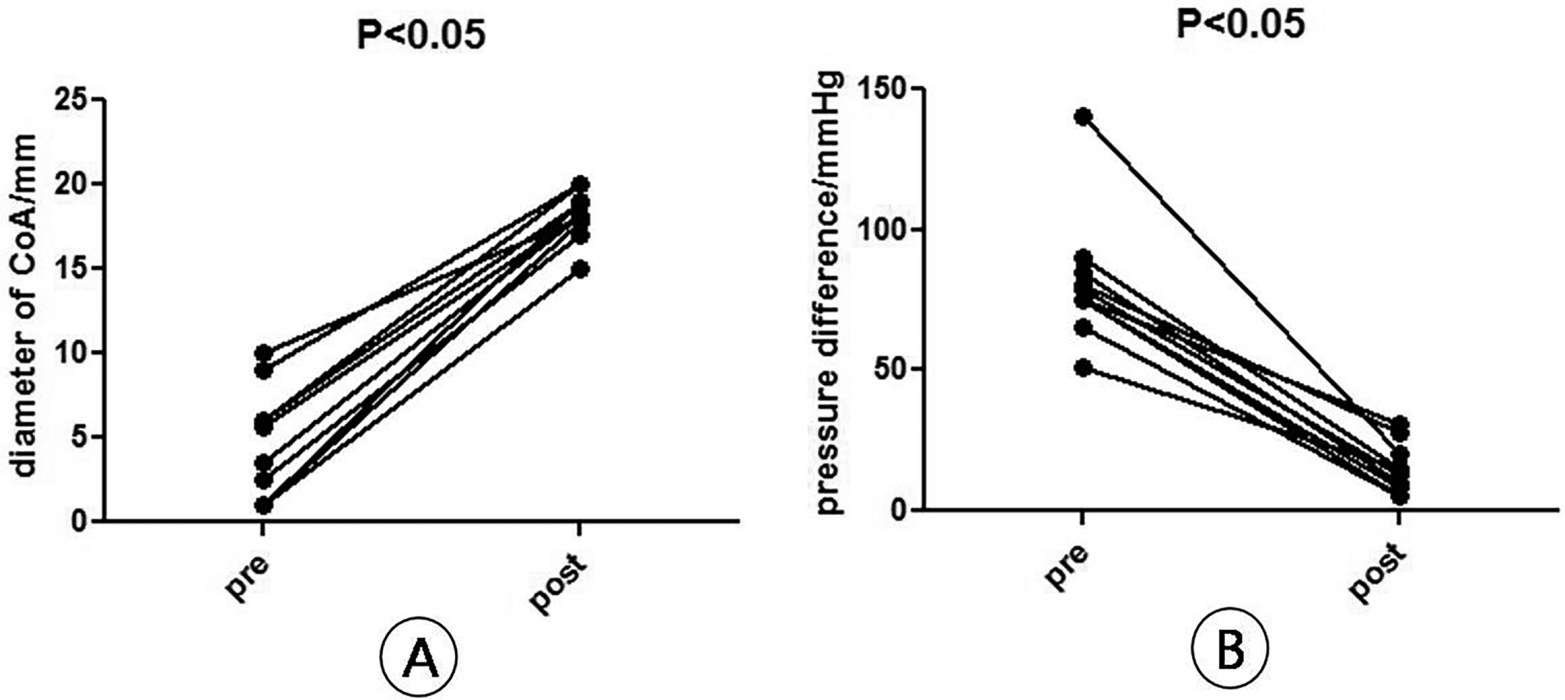
Diameter and pressure difference **(B)** at the narrowest point of arch constriction before and after surgery. **(A)** Diameter of CoA, *n* = 10, means ± SD, pre vs. post, *P* < 0.05; **(B)** pressure difference of CoA, *n* = 10, means ± SD, pre vs. post, *P* < 0.05.

All the patients with CoA were successfully implanted with covered stents. The narrowest mean diameter of CoA increased from (4.35 ± 2.61) mm before operation to (16.84 ± 1.99) mm immediately after operation (*P* < 0.05), and the mean transconstrictive systolic pressure difference decreased from (81.29 ± 18.72) mmHg before operation to (15.52 ± 7.47) mmHg after operation (*P* < 0.05). The mean systolic blood pressure of the right upper limb decreased from (182.05 ± 38.99) mmHg preoperatively to (141.95 ± 32.11) mmHg postoperatively (*P* < 0.05); the mean systolic blood pressure of the lower limb increased from (121.52 ± 27.84) mmHg preoperatively to (131.81 ± 32.39) mmHg postoperatively (*P* < 0.05) (Figure 6, Table 1). A total of two patients with VSD and PDA underwent interventional occlusion at the same time, and the shunt disappeared completely after operation. All patients had good healing of puncture points, no subcutaneous hematoma and vascular injury, no aortic dissection, aneurysm, pseudoaneurysm and other manifestations. After 12 months of follow-up, the clinical symptoms of the patients were relieved obviously, and the difference of systolic pressure between upper and lower limbs was less than 20 mmHg. Postoperative re-examination of aortic CTA showed that the stent was in good position and shape, and no dissection or aneurysm occurred (Figures 6, 7).

**Table 1:** Information about the balloon and stent used by the patient.

Patient	Sex (male/famale)	Age (years)	BIB balloon	CP stent
1	M	17Y	16.0 mm × 3.0 cm	3.4 cm
2	F	48Y	18.0 mm × 4.5 cm	3.4 cm
3	M	63Y	20.0 mm × 4.5 cm	3.9 cm
4	M	38Y	16.0 mm × 3.0 cm	3.4 cm
5	F	28Y	18.0 mm × 4.5 cm	3.4 cm
6	M	21Y	16.0 mm × 4.5 cm	3.9 cm
7	M	28Y	18.0 mm × 4.5 cm	3.4 cm
8	M	38Y	18.0 mm × 4.5 cm	3.9 cm
9	M	40Y	20.0 mm × 4.5 cm	3.4 cm
10	M	21Y	16.0 mm × 3.0 cm	3.4 cm

## Discussion

CoA refers to congenital coarctation of the aorta near the ductus arteriosus or ductal ligament, accounting for 5%–8% of congenital heart disease. Since 1991, O'Laughlin et al. (7) successfully treated one case of aortic constriction with balloon dilation stent for the first time, endovascular therapy has received widespread praise with the continuous development of interventional techniques. Interventional treatment for CoA includes balloon angioplasty (BA) and stent implantation (SI). In 2008, the American College of Cardiology and the College of Cardiology (ACC/AHA) guidelines (5) recommended interventional treatment for adult CoA, including: ① narrowing of the proximal and distal arterial peak pressure difference ≥20 mmHg, or ≤20 mmHg with significant narrowing or collateral blood flow imaging evidence; ② Recurrent or discontinuous narrowing with arterial pressure difference ≥20 mmHg. BA uses balloon dilation to expand the vascular lumen, which is simple to operate and effective, but can damage the intima and some media of the narrowed segment of the aortic wall. The use of a single BA to treat fibrous scar tissue in CoA cases can cause the narrowing segment to expand and retract, resulting in a higher incidence of restenosis (10%–15%) and the possibility of aneurysm formation. The incidence is higher in children and infants with CoA cases (11), and its application is limited. Compared with BA, SI uses intravascular stents to dilate narrowed arteries, and the continuous support of the stent on the vascular wall can effectively prevent the occurrence of restenosis. However, the problem of complications such as dissection and aneurysm caused by the injury of the aortic wall during bare stent placement still exists. For SI complications, Golden and Hellenbran (12) reviewed 565 cases of CoA stent placement from 17 institutions between 2002 and 2007, according to the Congenital Cardiovascular Interventional Study Consortium (CCISC). Among them, the pressure gradient after stent placement was less than 20 mmHg, accounting for 97% of all cases 9%. The incidence of acute complications was 81/565 (14.3%), including aneurysm formation (6/565, 1.1%), intimal tear (8/565, 1.4%), interlayer (9/565, 1.6%), bracket displacement (28/565, 5%), airbag rupture (13/565, 2.3%) and peripheral vascular complications including cerebrovascular accidents (17/565, 3.1%). Therefore, postoperative restenosis is a problem that needs to be considered. After the successful treatment of aortic aneurysm with CP stent, it was introduced into the interventional treatment of aortic constriction, effectively solving the problem of complications such as dissection and aneurysm caused by damage to the aortic wall during percutaneous balloon dilation and bare stent placement, significantly improving the safety of stent placement, and expanding the treatment indications for CoA (13). The NuMED CP stent is a novel stent system specifically designed for the treatment of aortic constriction in adolescents and adults. It belongs to the balloon expansion stent, mainly composed of NuMed BIB balloon and NuMed CP stent. BIB balloon is a dual balloon catheter (BIB catheter), and the CP stent is made of platinum material and covered with polytetrafluoroethylene membrane. The CP stent is not only beneficial for preventing restenosis, but its characteristics also include: high strength of the stent after expansion, strong plasticity of the alloy material, and small pressure on the aortic wall due to blunt edges; Large expandable range and low shortening rate; Previous balloon dilation of the stent can lead to a trumpet shaped opening at the proximal and distal ends, which can easily damage the aortic wall. Additionally, during the dilation process, the stent may move, resulting in inaccurate or displaced positioning of the stent. The BIB balloon is used to expand both the inner and outer balloons. Even with strong and high-pressure expansion, the stent will not have a small shape in the middle and a large shape at both ends, which helps with stent positioning. When the stent position is found to be unsatisfactory during internal balloon expansion, the stent position can be further adjusted to reduce the occurrence of complications. This reduces the probability of damage to blood vessels and balloons. Due to the PTFE membrane covering the stent, complications such as local aortic aneurysm or aortic dissection can be effectively avoided. At present, the CP balloon expandable CP stent has been widely used in clinical practice (6).

The results of this group of cases show that all 10 patients have successfully undergone CP stent placement surgery, and CP stents have good short-term and medium-term effects. Postoperative follow-up CTA showed good stent shape and position, and no serious complications such as stent displacement, rupture, restenosis, aortic dissection, or aneurysm were observed. CP stents pose a risk of blocking arterial blood flow perfusion in the aortic branch. Blocking the spinal artery can lead to serious neurological complications, such as paraplegia. However, the spinal artery usually originates below the level of the descending aorta diaphragm (beyond the 9th thoracic vertebra, >90% or more), so spinal artery occlusion cannot occur. In addition, when the hypoplasia of the aortic arch affects the left subclavian artery, CP stents can be used in combination with bare stents. The limitation of using CP stents for the treatment of aortic constriction is that the existing stents and pushers are 8–12 F, and the requirement for femoral artery diameter is not suitable for children. Even in older children, sheaths within this size range can cause damage to the femoral artery. Therefore, technological improvements in the delivery system will expand the applicability of stent therapy for aortic constriction. For young patients, due to the continued growth and development of the aorta, stent placement can cause relative restenosis of the stent segment. Therefore, research on biodegradable scaffolds and growable scaffolds will solve this problem. The former can gradually degrade automatically, eliminating the shortcomings of metal scaffolds that do not grow with growth and development. It can also protect damaged intima and reduce complications. The latter is composed of two symmetrical half stainless steel stents connected by absorbable wires to form a complete stent, which can be repeatedly expanded, while often requires multiple percutaneous balloon dilation or re stent placement after surgery (14). CP stent replacement is a safe and effective method for treating aortic stenosis in adolescents and adults. The short-term results are satisfactory. However, the long-term effects require long-term follow-up observation and more case studies.

The use of 3D models for individual patient model reconstruction in preoperative planning and surgical planning has been applied in structural heart disease surgery (15–18). 3D printing technology can facilitate the smooth completion of complex surgeries, especially in dealing with challenging interventional treatment cases. There have been many successful cases of using 3D printing technology to assist surgical simulation and preoperative evaluation in the cardiovascular field both domestically and internationally (19, 20). This study utilized 3D printing technology to create models of the entire heart and aorta. Prior to surgery, the patient's anatomical structure of the heart was fully understood. A 3D printing model that accurately replicated the anatomical structure of the patient's lesion site can provide a visual reference for the surgeon before surgery, and together with the results of ultrasound and CT examinations, it can be used as preoperative multimodal imaging evaluation data. At the same time, the 3D printing model is also a tool for surgical simulation, including the location and angle of the stent release, *in vitro* measurement and simulation of corresponding indicators, and combined with preoperative ultrasound and other imaging measurement tools to achieve the role of preoperative multimodal imaging evaluation.

This study validated the effectiveness and safety of interventional treatment for CoA, while also reflecting the role of 3D printing technology in assisting preoperative and postoperative imaging evaluation. With the rapid development of imaging and computer technology, the accuracy and simulation level of cardiovascular 3D printing are constantly improving. The 3D printing technology compensates for the shortcomings of traditional imaging technology in displaying complex structures, especially 3D reconstruction, which enables cardiovascular physicians to have a more intuitive understanding of the morphology and structure of disease changes, bringing new ideas for the precise treatment of heart disease patients (21–23).

## Study limitations

To gain a better insight into the auxiliary role of 3D printing technology in CoA disease, population-based multicenter case-control studies with a prospective design are needed.

## Conclusions

To sum up, the mastery of indications and the clinical experience of the surgeon are very important for the success of the operation as well as the role of 3D printing technology in assisting preoperative and postoperative imaging evaluation. Percutaneous balloon dilatation covered stent implantation for CoA in adolescents and adults has a definite short-term and medium-term effect, high success rate and few related complications. The long-term effect needs further study.

## Data Availability

The original contributions presented in the study are included in the article/Supplementary Material, further inquiries can be directed to the corresponding authors.
